# Supplementary figures and images for: Comparison of the bleaching susceptibility of coral species by using minimal samples of live corals
Source: PeerJ. 2022 Jan 26;10:e12840. doi: 10.7717/peerj.12840 (PMC8800388; doi:10.7717/peerj.12840)

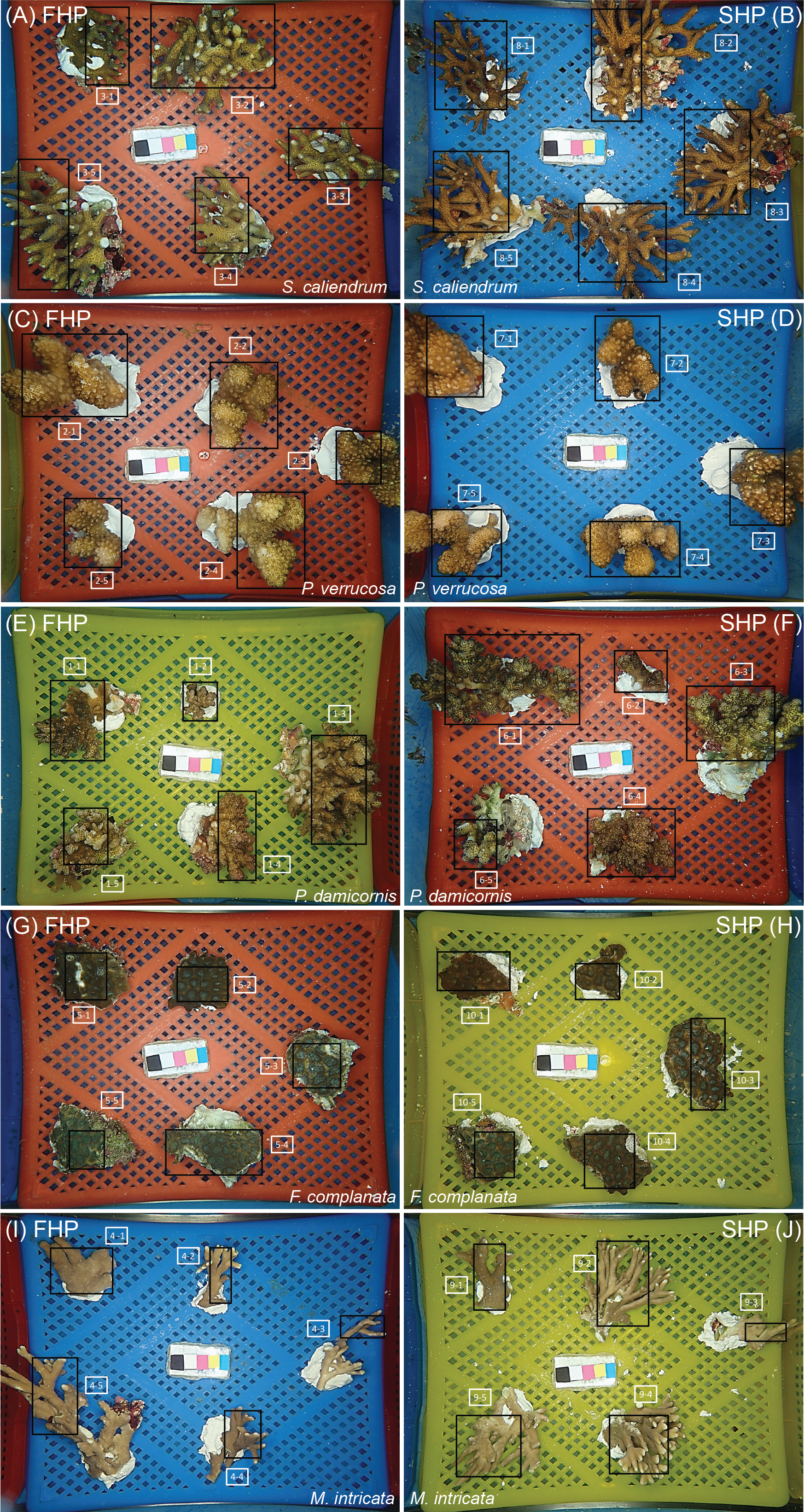

Supplement: Supplemental Information 1 — Numbers in white boxes represent colony replicates. The region of interest in the image for grayscale calculation is indicated by the black square, and the same region was used to calculate grayscale changes in coral fragments during heating experiment with FHP (ACEGI) and SHP (BDFHJ). (AB): Seriatopora caliendrum; (CD): Pocillopora verrucosa; Pocillopora damicornis (EF); (GH): Favites complanata; (IJ): Millepora intricata. Color strip in the middle of each photograph was used to standardize the grayscale value of each target area. [file peerj-10-12840-s001.png]
