# Supplementary material for: Comparison of the bleaching susceptibility of coral species by using minimal samples of live corals
Source: PeerJ. 2022 Jan 26;10:e12840. doi: 10.7717/peerj.12840 (PMC8800388; doi:10.7717/peerj.12840)
Supplement: Supplemental Information 2 [file peerj-10-12840-s002.pdf]

Step 1: Open an image file in Photoshop and mark the fixed area for grayscale estimation.

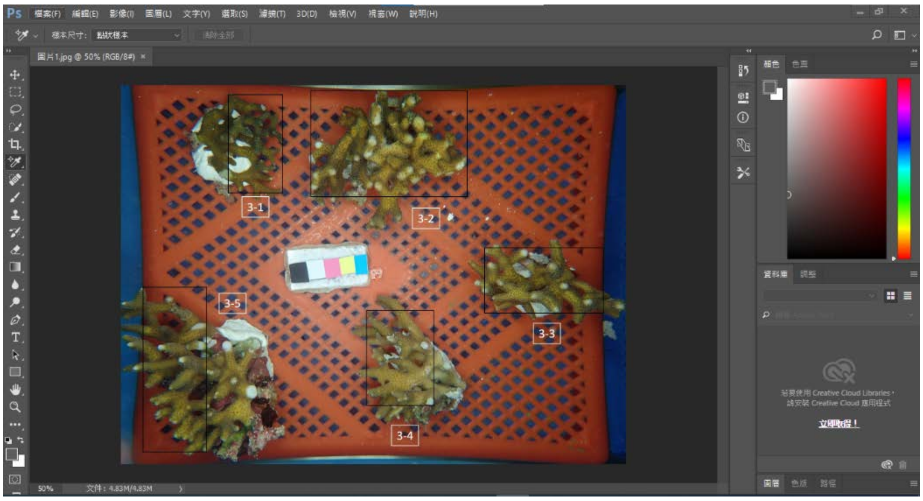

Step 2: Convert color image into grayscale image.

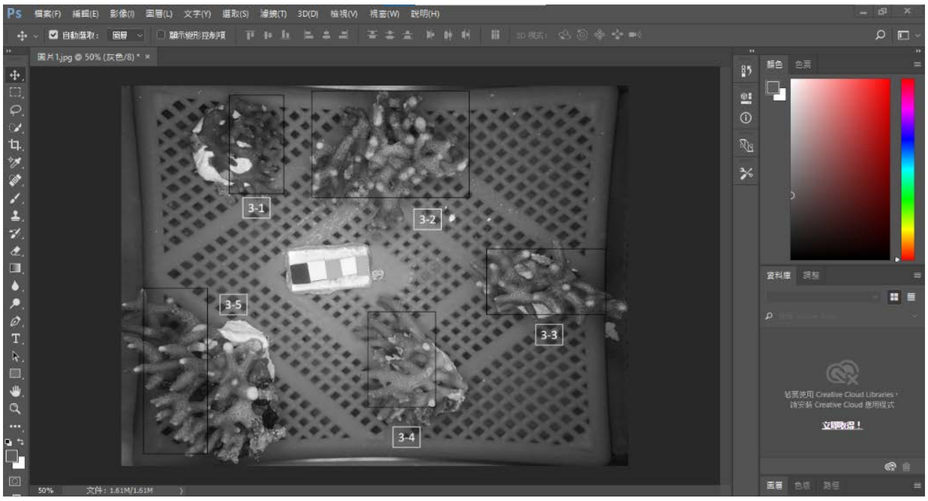

Step 3: Box the square with “rectangle select” and average the grayscale in the selected area.

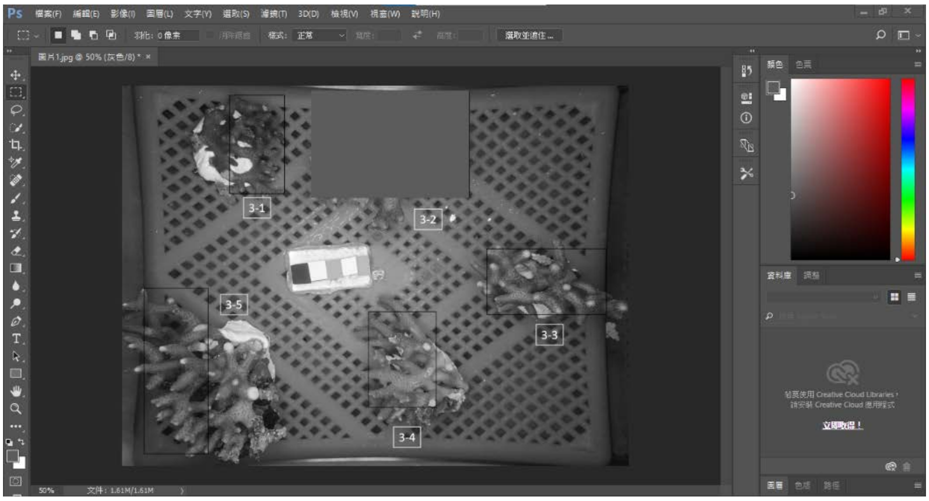

Step 4: Use “color selecting tool” to target the area for grayscale estimation (estimated grayscale is 77% in this case)

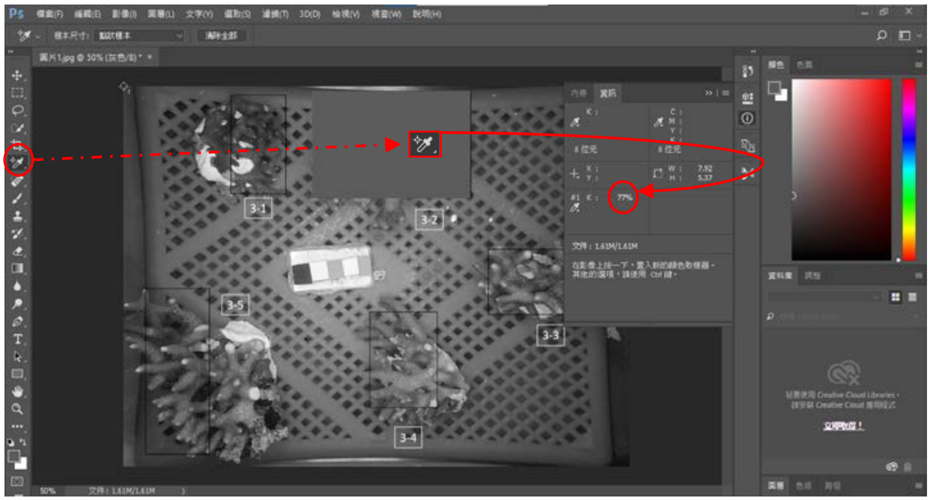

Step 5: Repeat the procedure from step 3 to 4 to estimate grayscale of standard black and white color strips (black: 80% and white: 7% in this case).

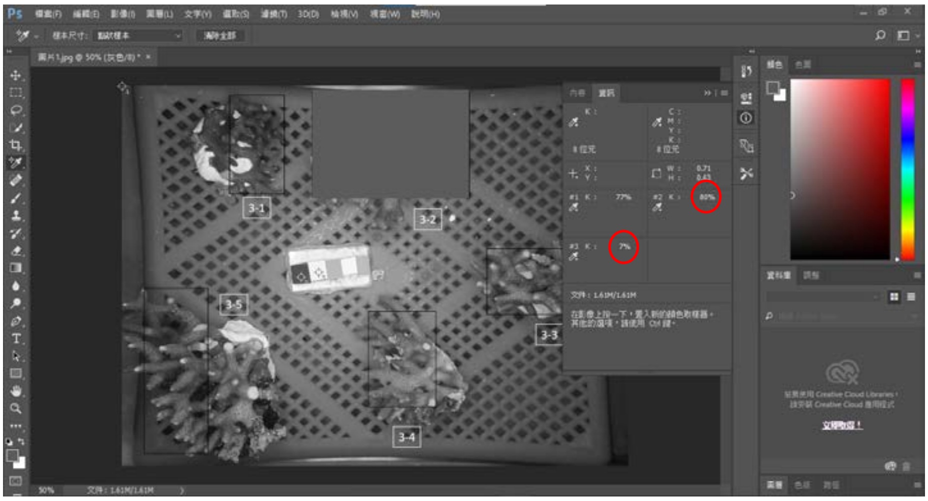

Step 6: Estimate G_0 of a coral fragment at a time point.

G_C (grayscale of the coral fragment) = 77%

G_B (grayscale of standard black color strip) = 80%

G_W (grayscale of standard white color strip) = 7%

Then, above values are applied into the equation, $G_0 = \left(\frac{G_C - G_W}{G_B - G_W} \right)$, which results in that G_0 of the coral fragment is (77-7)/(80-7) *100%= 95.9%.
