# Supplementary material for: Comparison of the bleaching susceptibility of coral species by using minimal samples of live corals
Source: PeerJ. 2022 Jan 26;10:e12840. doi: 10.7717/peerj.12840 (PMC8800388; doi:10.7717/peerj.12840)
Supplement: Supplemental Information 3 — The relative grayscale values of each coral replicate during heat treatment were fitted onto a modified Gaussian equation to describe the bleaching process. Modified Gaussian equation: f(X) = y0 + a × exp( − 0.5 × abs[(X − x0)/b]c). [file peerj-10-12840-s003.docx]

| *Seriatopora caliendrum* | 1^o^C / day | | | | | | | |
| --- | --- | --- | --- | --- | --- | --- | --- | --- |
|  |  | No.3-1 | No.3-2 | No.3-3 | No.3-4 | No.3-5 | mean | sd |
|  | R^2^ | 0.9825 | 0.9906 | 0.9831 | 0.9860 | 0.9812 | 0.9847 | 0.0037 |
|  | a | 104.6 | 99.10 | 103.5 | 107.9 | 102.3 |  |  |
|  | b | 13.24 | 26.86 | 4599 | 2.62 | 6.77 |  |  |
|  | c | 10.55 | 28.36 | 3438 | 1.91 | 5.52 |  |  |
|  | x_0_ | -7.22 | -21.31 | -4593 | 1.68 | -1.40 |  |  |
|  | y_0_ | -3.67 | 0.54 | -1.47 | -2.64 | -1.76 |  |  |
|  | 1^o^C / 3 days | | | | | | | |
|  |  | No.8-1 | No.8-2 | No.8-3 | No.8-4 | No.8-5 | mean | sd |
|  | R^2^ | 0.9892 | 0.9924 | 0.9924 | 0.9884 | 0.9918 | 0.9908 | 0.0019 |
|  | a | 100.1 | 98.84 | 96.99 | -95.25 | 96.35 |  |  |
|  | b | 3.40 | 6.80 | 75455 | 4.28 | 5.46 |  |  |
|  | c | 25.42 | 10.73 | 143144 | 7.64 | 7.65 |  |  |
|  | x_0_ | 3.71 | 5.53 | -75446 | 12.33 | 3.97 |  |  |
|  | y_0_ | -0.94 | -1.27 | -0.25 | 95.95 | 1.54 |  |  |
|  | | | | | | | | |
| *Pocillopora verrucosa* | 1^o^C / day | | | | | | | |
|  |  | No.2-1 | No.2-2 | No.2-3 | No.2-4 | No.2-5 | mean | sd |
|  | R^2^ | 0.9837 | 0.9860 | 0.9842 | 0.9861 | 0.9920 | 0.9864 | 0.0033 |
|  | a | 100.6 | 98.25 | 98.88 | 97.41 | 97.48 |  |  |
|  | b | 3.68 | 1646 | 4.04 | 1384 | 4.34 |  |  |
|  | c | 4.53 | 4057 | 7.19 | 3336 | 9.04 |  |  |
|  | x_0_ | 2.62 | -1639 | 2.49 | -1377 | 2.45 |  |  |
|  | y_0_ | -0.28 | -0.14 | 1.05 | -1.22 | 0.66 |  |  |
|  | 1^o^C / 3 days | | | | | | | |
|  |  | No.7-1 | No.7-2 | No.7-3 | No.7-4 | No.7-5 | mean | sd |
|  | R^2^ | 0.9931 | 0.9849 | 0.9677 | 0.9907 | 0.9774 | 0.9828 | 0.0104 |
|  | a | -99.39 | 101.9 | 89.80 | 96.31 | 94.03 |  |  |
|  | b | 7.22 | 3.40 | 64900 | 9.92 | 1469 |  |  |
|  | c | 3.64 | 6.48 | 66804 | 6.51 | 3275 |  |  |
|  | x_0_ | 16.75 | 3.22 | -64886 | 6.29 | -1455 |  |  |
|  | y_0_ | 99.83 | -0.82 | -0.65 | 2.01 | -0.78 |  |  |
|  | | | | | | | | |
| *Pocillopora damicornis* | 1^o^C / day | | | | | | | |
|  |  | No.1-1 | No.1-2 | No.1-3 | No.1-4 | No.1-5 | mean | sd |
|  | R^2^ | 0.9845 | 0.9924 | 0.9931 | 0.9897 | 0.9879 | 0.9895 | 0.0035 |
|  | a | 94.46 | 101.8 | 95.94 | 97.55 | 97.55 |  |  |
|  | b | 998.3 | 4.53 | 4.24 | 4.15 | 4.25 |  |  |
|  | c | 3514 | 29.43 | 21.62 | 6.15 | 12.10 |  |  |
|  | x_0_ | -991.2 | 3.66 | 2.99 | 2.06 | 2.78 |  |  |
|  | y_0_ | 1.42 | 0.04 | 1.42 | 1.96 | 3.37 |  |  |
|  | 1^o^C / 3 days | | | | | | | |
|  |  | No.6-1 | No.6-2 | No.6-3 | No.6-4 | No.6-5 | mean | sd |
|  | R^2^ | 0.9939 | 0.9767 | 0.9910 | 0.9916 | 0.9625 | 0.9831 | 0.0134 |
|  | a | 99.67 | 119.1 | 99.65 | 96.32 | 102.2 |  |  |
|  | b | 53524 | 30929 | 7388 | 8.69 | 5.68 |  |  |
|  | c | 24886 | 5356 | 4256 | 8.70 | 7.74 |  |  |
|  | x_0_ | -53512 | -30917 | -7373 | 4.80 | 5.10 |  |  |
|  | y_0_ | -0.36 | -13.66 | -0.75 | 0.44 | -1.06 |  |  |
|  | | | | | | | | |
| *Favites complanata* | 1^o^C / day | | | | | | | |
|  |  | No.5-1 | No.5-2 | No.5-3 | No.5-4 | No5-5 | mean | sd |
|  | R^2^ | 0.9897 | 0.9978 | 0.9969 | 0.9977 | 0.9944 | 0.9953 | 0.0034 |
|  | a | 97.61 | 98.00 | 97.12 | 97.42 | 96.23 |  |  |
|  | b | 3272 | 3525 | 1507 | 4.63 | 4.93 |  |  |
|  | c | 3716 | 7562 | 4257 | 8.88 | 14.15 |  |  |
|  | x_0_ | -3263 | -3516 | -1500 | 2.99 | 3.59 |  |  |
|  | y_0_ | -0.92 | 0.17 | 1.68 | 1.19 | 2.09 |  |  |
|  | 1^o^C / 3 days | | | | | | | |
|  |  | No.10-1 | No.10-2 | No.10-3 | No.10-4 | No.10-5 | mean | sd |
|  | R^2^ | 0.9935 | 0.9952 | 0.9963 | 0.9902 | 0.9890 | 0.9928 | 0.0032 |
|  | a | 97.73 | 99.72 | 97.27 | 97.37 | 97.96 |  |  |
|  | b | 15915 | 34.40 | 5463 | 11313 | 7580 |  |  |
|  | c | 6250 | 9.54 | 5350 | 4397 | 3842 |  |  |
|  | x_0_ | -15893 | -17.62 | -5446 | -11293 | -7560 |  |  |
|  | y_0_ | -0.40 | -0.50 | -0.17 | -1.45 | -1.14 |  |  |
|  | | | | | | | | |
| *Millepora* *intricata* | 1^o^C / day | | | | | | | |
|  |  | No.4-1 | No.4-2 | No.4-3 | No.4-4 | No4-5 | mean | sd |
|  | R^2^ | 0.9942 | 0.9897 | 0.9768 | 0.9930 | 0.9935 | 0.9894 | 0.0073 |
|  | a | 100.7 | -107.8 | 116.7 | 109.3 | -102.1 |  |  |
|  | b | 9.39 | 10.43 | 7.20 | 7.86 | 11.56 |  |  |
|  | c | 2.60 | 2.81 | 1.80 | 1.97 | 2.77 |  |  |
|  | x_0_ | 4.30 | 26.44 | 5.29 | 4.70 | 28.51 |  |  |
|  | y_0_ | 1.45 | 104.0 | -2.77 | -1.43 | 101.81 |  |  |
|  | 1^o^C / 3 days | | | | | | | |
|  |  | No.9-1 | No.9-2 | No.9-3 | No.9-4 | No.9-5 | mean | sd |
|  | R^2^ | 0.9567 | 0.9509 | 0.9326 | 0.9716 | 0.9786 | 0.9581 | 0.0181 |
|  | a | 224.8 | 778.5 | 111001 | 148.2 | 92078 |  |  |
|  | b | 17.63 | 30.16 | 311.1 | 16.22 | 256.09 |  |  |
|  | c | 2.80 | 2.38 | 2.07 | 3.20 | 2.20 |  |  |
|  | x_0_ | 11.42 | 12.43 | 13.66 | 9.76 | 12.89 |  |  |
|  | y_0_ | -108.1 | -657.4 | -110850 | -39.81 | -91932 |  |  |
