# Supplementary material for: Comparison of the bleaching susceptibility of coral species by using minimal samples of live corals
Source: PeerJ. 2022 Jan 26;10:e12840. doi: 10.7717/peerj.12840 (PMC8800388; doi:10.7717/peerj.12840)
Supplement: Supplemental Information 4 — The data were calculated from subtracting 100% by the highest relative grayscale obtained during heating process, and expressed as Mean ± SD (n=5). [file peerj-10-12840-s004.docx]

|  | Increase in relative grayscale (%) | | | | |
| --- | --- | --- | --- | --- | --- |
|  | *S. caliendrum* | *P. verrucosa* | *P. damicornis* | *F. complanata* | *M.* *intricata* |
| FHP | 4 ± 2 | 9 ± 3 | 8 ± 2 | 2 ± 1 | 10 ± 6 |
| SHP | 8 ± 2 | 7 ± 3 | 9 ± 4 | 4 ± 2 | 40 ± 20 |
