# Supplementary material for: Comparison of the bleaching susceptibility of coral species by using minimal samples of live corals
Source: PeerJ. 2022 Jan 26;10:e12840. doi: 10.7717/peerj.12840 (PMC8800388; doi:10.7717/peerj.12840)
Supplement: Supplemental Information 5 [file peerj-10-12840-s005.docx]

| ANOVA Table | | | | | |
| --- | --- | --- | --- | --- | --- |
| FHP | | | | | |
| BTI_10_ | | | | | |
|  | SS | DF | MS | F | P |
| Within | 85.296 | 4 | 21.324 | 50.072 | 3.840E-10 |
| Between | 8.517 | 20 | 0.426 |  |  |
| Total | 93.814 | 24 |  |  |  |
| BTI_30_ | | | | | |
| Within | 157.587 | 4 | 39.397 | 105.616 | 3.756E-13 |
| Between | 7.460 | 20 | 0.373 |  |  |
| Total | 165.047 | 24 |  |  |  |
| BTI_50_ | | | | | |
| Within | 260.929 | 4 | 65.232 | 181.037 | 2.110E-15 |
| Between | 7.206 | 20 | 0.360 |  |  |
| Total | 268.135 | 24 |  |  |  |
| SHP | | | | | |
| BTI_10_ | | | | | |
|  | SS | DF | MS | F | P |
| Within | 743.609 | 4 | 185.902 | 25.369 | 1.369E-07 |
| Between | 146.561 | 20 | 7.328 |  |  |
| Total | 890.170 | 24 |  |  |  |
| BTI_30_ | | | | | |
| Within | 894.813 | 4 | 223.703 | 36.783 | 5.902E-09 |
| Between | 121.632 | 20 | 6.082 |  |  |
| Total | 1016.445 | 24 |  |  |  |
| BTI_50_ | | | | | |
| Within | 1054.014 | 4 | 263.504 | 43.848 | 1.260E-09 |
| Between | 120.190 | 20 | 6.009 |  |  |
| Total | 1174.204 | 24 |  |  |  |
