# Supplementary material for: Comparison of the bleaching susceptibility of coral species by using minimal samples of live corals
Source: PeerJ. 2022 Jan 26;10:e12840. doi: 10.7717/peerj.12840 (PMC8800388; doi:10.7717/peerj.12840)
Supplement: Supplemental Information 6 — The variations were estimated by coefficient of variations (CV). [file peerj-10-12840-s006.docx]

|  | CV (%) | | | | | | | | |
| --- | --- | --- | --- | --- | --- | --- | --- | --- | --- |
|  | *S. caliendrum* | | *P. verrucosa* | *P. damicornis* | | *F. complanata* | | | *M.* *intricata* |
| FHP |  | |  |  | |  | | |  |
| BTI_10_ | 11.8 | | 7.0 | 14.6 | | 10.8 | | | 4.7 |
| BTI_20_ | 12.6 | | 6.1 | 12.3 | | 10.2 | | | 2.5 |
| BTI_30_ | 12.2 | | 5.1 | 10.9 | | 10.1 | | | 3.0 |
| BTI_40_ | 11.3 | | 4.3 | 9.8 | | 10.2 | | | 3.3 |
| BTI_50_ | 10.2 | | 3.4 | 8.8 | | 10.4 | | | 3.3 |
|  | Mean ± SD = 8.3 ± 3.7 (n = 25) | | | | | | | | |
|  | CV (%) | | | | | | | | |
|  | *S. caliendrum* | *P. verrucosa* | | | *P. damicornis* | | *F. complanata* | *M.* *intricata* | |
| SHP |  | |  |  | |  | | |  |
| BTI_10_ | 21.0 | | 39.9 | 28.2 | | 15.6 | | | 8.6 |
| BTI_20_ | 21.6 | | 37.9 | 16.8 | | 13.1 | | | 6.8 |
| BTI_30_ | 21.9 | | 36.5 | 11.9 | | 12.1 | | | 5.5 |
| BTI_40_ | 22.1 | | 35.3 | 10.3 | | 11.5 | | | 4.5 |
| BTI_50_ | 22.2 | | 34.3 | 10.6 | | 11.2 | | | 3.7 |
|  | Mean ± SD =24.7 ± 10.1 (n = 15) | | | | | Mean ± SD = 9.3 ± 4.0  (n =10) | | | |
